# A New Variant Mutation in *SKIV2L* Gene in Case of Trichohepatoenteric Syndrome

**DOI:** 10.3390/pediatric12030021

**Published:** 2020-10-26

**Authors:** Ziad A. Taher, Saeed Alzahrani, Abdullah Alsaghir, Faris Nouh, Mesbah Alshumrani

**Affiliations:** 1 Department of Medicine, Ministry of National Guard Health Affair, King Saud Bin Abdulaziz University for Health Sciences, COM-WR, King Abdullah International Medical Research Center, Jeddah 21423, Saudi Arabia; saeed-1412-@hotmail.com (S.A.); aymalsaghir@gmail.com (A.A.); faris.nouh@hotmail.com (F.N.); 2 Department of Pediatrics, Ministry of National Guard Health Affair, Jeddah 21423, Saudi Arabia; mesbah.alshumrani@gmail.com

**Keywords:** trichohepatoenteric syndrome, intractable diarrhea, TPN

## Abstract

Trichohepatoenteric syndrome is an autosomal recessive genetic disease with an estimated prevalence of 1:100,000. The mutation of the disease is placed either in *SKIV2L* or *TTC37* genes. The onset of presentation is variable, but symptoms usually start with intractable diarrhea associated with woolly hair abnormality, immune dysfunction, and sometimes hepatic abnormality. This case is of a 10-month-old girl who was born at 37 + 2 weeks due to symmetrical intrauterine growth restriction (IUGR), with a low birth weight (1320 g). It was noticed during her stay in NICU that she had excessive diarrhea on day 8. Gastroenterology suggested starting an extensively-hydrolyzed formula, but no improvement noticed. The multidisciplinary teams decided to order whole-exome sequencing analysis after excluding diarrhea causes. The analysis detected a new variant mutation (*c.1297C* > *T*) p. (Arg433Cys). To our knowledge, this is the first time detected in a homozygous state in the *SKIV2L* gene, as this variant mutation has not been described in any previous literature. Our case was managed mainly by total parenteral nutrition. The patient responded to the treatment appropriately.

## 1. Introduction

Trichohepatoenteric syndrome (THES; OMIM:600478) or syndromic diarrhea (SD), also known as phenotypic diarrhea (PD), is a congenital rare and severe Mendelian autosomal recessive inherited genetic disease with an estimated prevalence of 1:1,000,000. Trichohepatoenteric syndrome characterized enteropathy, presenting mainly with early-onset of severe diarrhea, which subsequently leads to failure to thrive (FTT) and hair abnormality [1]. It was first described in 1982 by Stankler et al. and explored more in 1994 by D Girault et al. as a clinical entity characterized by severe infant diarrhea combined with physical abnormalities and deficiencies of the immune system [2,3]. 

THES is a rare genetic disorder associated with pathogenic variants mutation in super killer viralicidic activity 2 (*SKIV2L*) or tetratricopeptide repeat domain-containing protein 37 (*TTC37*), both encode for proteins of a super killer complex, which is part of the RNA exome [4]. THES typically presents in the neonatal period with intractable diarrhea, almost always requiring parenteral nutrition, and subsequent poor weight gain and failure to thrive, woolly and brittle hair, and facial dysmorphisms [5]. Other features are immunodeficiency, skin abnormalities, liver disease, mild intellectual disability, and rare congenital heart disease [6,7]. 

Recently, increasing attention has been paid to the relationship between *TTC37* or *SKIV2L* gene mutations and the pathogenesis of very early-onset inflammatory bowel disease. Histopathological features of small intestine biopsy are nonspecific villous atrophy, with or without mixed inflammatory bowel disease resembling colitis. Other characteristic features of THES include hepatic involvement, which can develop irrespective of parenteral nutrition. Fabre et al. reported that 12 of 22 (55%) patients with THES had liver disease, 9 of 18 (50%) had hepatic cirrhosis, and 4 of 17 (24%) had hepatic hemosiderosis, indicating that iron overload may contribute to THES pathogenesis [4]. It is difficult to clearly define the type of immunodeficiency associated with THES, since literature reports are inconsistent. However, the low serum concentration of immunoglobulins and the absence of humoral immune responses to vaccination are the most frequently reported conditions. Patients with THES usually die in early childhood due to severe infection or liver cirrhosis, and early diagnosis is vital for optimal management and to improve outcomes for THES patients [8].

Up to our knowledge, a few cases have been reported for THES with genetic confirmation of the disease. In Asia, there are two reported cases with genetic confirmation, and there is no report for such a case in the middle east.

Here we report the first case of THES in the middle east with confirmation by genetic testing. Moreover, it is the first case to be reported worldwide with a unique mutation in the *SKIV2L* gene.

## 2. Case Report

The reported case is about a 10-month-old girl of an Arabic ethnicity who suffered from intractable diarrhea and failure to thrive.

She was born at 37 + 2 weeks, emergent CS due to abnormal Doppler with symmetrical IUGR, birth weight 1.320 Kg (below the 3rd centile), with a head circumference of 29 cm (<3rd centile), and height of 37 cm (<3rd centile). She was the 2nd born child of a first-degree related parent. The older child is a 6-year-old boy who has no medical issues.

Mother was not following regularly during pregnancy; her serology was done late and revealed negative results. The antenatal ultrasound revealed evidence of IUGR (EFW of 2%) and oligohydramnios. The baby was born with an Apgar score of 8 and 9 but later developed respiratory distress, requiring intubation and admission to NICU for a few days. Additionally, she was diagnosed with G6PD deficiency and small PDA.

After eight days of NICU admission, the case was referred to the gastroenterology team after noticing unexplained chronic diarrhea. She was on an extensively - hydrolyzed formula. Initially, she did not gain weight but noticed to gain weight when she was NPO (nothing by mouth) on TPN (Total parenteral nutrition).

Multiple work up was sent to rule out different possible causes of chronic diarrhea at this age, and all were negative. Subsequently, she was seen by the genetic team, and the Whole Exon Sequence (WES) genetic test were sent. Later, the patient diagnosis was confirmed by WES analysis as a case of THES 2. WES analysis showed the detection of new variant mutation (*c.1297C > T*) p. (*Arg433Cys*). Up to our knowledge, this variant mutation has not been described in any previous literature so far. Based on Bioscientia Human Genetics database, this variant is found in 0.0038% of the general population (8 heterozygous, 0 homozygous), but this is the first time to be detected in a homozygous state in *SKIV2L* gene. This mutation leads to the amino acid exchange mentioned above, which subsequently manifested as autosomal recessive THES 2.

Trichohepatoenteric syndrome is treated symptomatically, meaning it is focused on treating the symptoms of the disease. Treating chronic diarrhea is often the biggest concern, as chronic diarrhea can prevent people with trichohepatoenteric syndrome from getting enough nutrients. 

**Figure 1:**
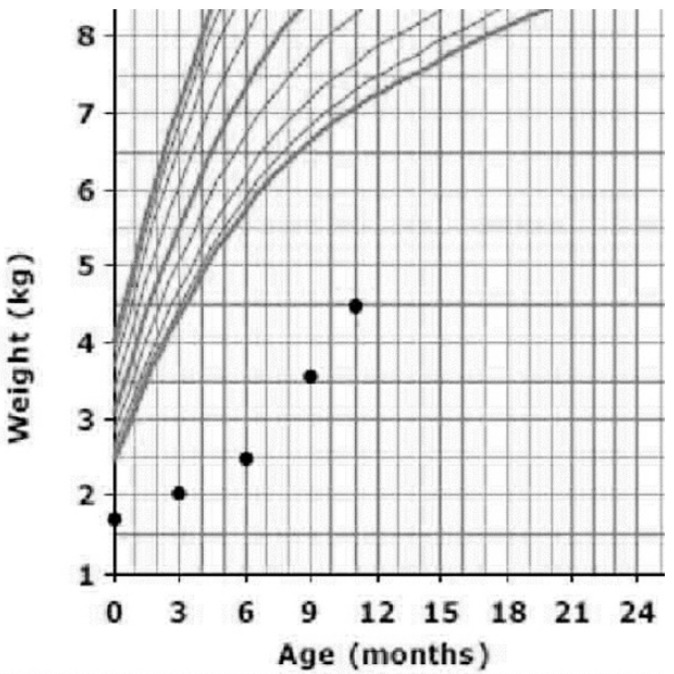
This graph shows the development of the patient before and after starting the management, this growth chart is for Saudi children and adolescents in the female population.

At the age of 3 months, after confirmation of the diagnosis, she was started on TPN started and lipid infusion over 20 h beside oral feeding of extensively hydrolyzed formula. Her weight improved from 1320 g at birth to 4500 g at the age of 10 months. Even though it is not the optimal weight, she is gaining weight better than previously (Figure 1).

At the age of 6 months, she had a bilateral inguinal hernia, which was surgically corrected without ant complication.

After starting the TPN, she was treated a couple of times for line related bacterial infections, otherwise no significant infections. She was screened for immune deficiency on different occasions; her result always within the normal range.

Currently, she has a global developmental delay. She cannot roll over, but she can lie in a prone position with the support of the forearm. She can reach an object near to her. Speech assessment showed her ability to say “dada”. Socially, she is interactive with her parents. Overall, her developmental age is around 3–5 months.

Unfortunately, liver disease or a weakened immune system resulting in severe infections cause about 50% of people affected by trichohepatoenteric syndrome to pass away during childhood. However, individuals that survive childhood may no longer require IV nutrition. Most people with trichohepatoenteric syndrome will be smaller than other people their age. The mild intellectual disability associated with trichohepatoenteric syndrome may require a person to have special classes in school.

## 3. Case Discussion

As long as THES is a rare syndrome, it can be easily missed ending up with the wrong diagnosis. Delaying the diagnosis will result in receiving the wrong management, that will affect his quality of life and worsen the prognosis.

In human, two genes have been shown to cause THES: *TTC37* and *SKIV2L* [9].

In Alexandre Fabre et al. they reviewed the literature of a total of 80 patients, 40 Patients with mutation of *TTC37*, 14 Patients with mutation of *SKIV2L*, one patient without mutation of *SKIV2L* or *TTC37*, and 25 patients not tested. This study shows that *SKIV2L* mutation is rare, which is the scenario in this study. 

Moreover, the most common symptoms are chronic diarrhea, hair abnormality with prevalence of liver abnormality in about half the reported cases [9]. Liver function in this patient was reassuring, which likely will have a better prognosis if compliant on the suggested plan. 

TPN manages most of the cases after the establishment of the diagnosis, though most of the cases have poor outcomes due to TPN complications, which is the only currently available treatment. An only a small percentage of the cases reach normal oral nutrition, after many years [6,9]. In our case, the patient is mainly using a TPN as the primary source of nutrition, and she is on extraoral extensively-hydrolyzed formula. She has no TPN-related complications apart from central line-related infections.

In the case of Zheng B et al., the case was complicated by inguinal hernia at the age of 3 months, in the 3rd-day post-hernia repair, the case was complicated more by severe lower respiratory tract infection, pleural effusion, ascites, and liver cirrhosis [10]. Differently, our case was only involved a developed bilateral inguinal hernia without post-op complications.

The onset of THES symptoms was variable in many studies. Frédéric Vély et al. noticed that the disease could start at birth or as late as after 335 days of delivery. Moreover, the birth weight was also was variable from 1000 g to 2950 g [11]. Compared to our case, the onset of symptoms was noted in the first eight days of life.

Skin abnormality and dysmorphism are common to appear in THES. Its prevalence in one of the literates was 7/8 and 6/8, respectively. However, this was not clear in our case, as the patient had woolly and brittle hair, but the skin is healthy and no dysmorphic features [11].

This study has two limitations. First, the status of parents regarding the mutation status is not known. Second, the Sanger method was not done due to the unavailability in the center of the study.

In conclusion, further knowledge is needed to understand and manage such rare and fatal syndrome. The early genetic confirmation of this syndrome and introducing of total parental nutrition could play a role in delaying complications and improved the outcome of such cases.
